# Recurrent Acrodysostosis-Related PKA RIα Mutant Reveals a Novel Mechanism of Aberrant PKA Deactivation^☆^

**DOI:** 10.1016/j.jmb.2025.169381

**Published:** 2025-08-09

**Authors:** Leonardo Della Libera, Karla Martinez Pomier, Madoka Akimoto, Ganesh S. Anand, Susan S. Taylor, Giuseppe Melacini

**Affiliations:** 1 Department of Chemistry and Chemical Biology, McMaster University, Hamilton, ON, Canada; 2 Department of Chemistry, Department of Biochemistry and Molecular Biology, and The Huck Institutes of the Life Sciences, The Pennsylvania State University, University Park, PA 16802, USA; 3 Department of Pharmacology and Department of Chemistry and Biochemistry, University of California, San Diego, La Jolla, CA 92037-0654, USA; 4 Department of Biochemistry and Biomedical Sciences, McMaster University, Hamilton, ON, Canada

**Keywords:** NMR, cAMP, PKA, allostery, dynamics

## Abstract

Protein kinase A (PKA) is essential in converting extracellular signals into tightly regulated cellular responses controlling vital processes such as growth, development, and gene expression. Activation of PKA is controlled by the binding of cAMP to the regulatory subunit of PKA (R). Several mutations in the ubiquitous RIα isoform of R cause Acrodysostosis 1 (ACRO), a disease characterized by resistance to thyroid-stimulating and parathyroid hormones leading to severe congenital malformations. This work examines the recurrent R366X truncation ACRO mutant, which exhibits severe PKA hypoactivation due to loss of sensitivity to cAMP and the impairment of allosteric networks. The R366X RIα mutant has been previously studied via X-ray crystallography, but the crystal structure only captured the inhibited state and showed minimal difference from the wild type structure. Additionally, previous studies only examined the effects of ACRO mutants on the activation cycle of PKA (*i.e*. sensitivity to cAMP binding). Here we focus on the less understood signal termination cycle. We hypothesize that R366X acts by perturbing dynamic intermediates relevant to the PKA deactivation cycle, which are not fully recapitulated by static structures. To test our hypothesis, we combined low- and high-resolution approaches for probing protein–ligand binging, mutant stability, and identifying regions exhibiting aberrant allosteric behaviors. Based on our results, we propose a novel mechanism whereby R366X not only impairs physiological PKA activation but also accelerates PKA deactivation by increasing the rate of phosphodiesterase-catalyzed cAMP hydrolysis to 5′-AMP. Our studies shed new light on the current understanding of PKA dysregulation and ACRO’s molecular etiology, outlining a multi-resolution experimental design which is transferable to other ACRO mutants.

## Introduction

**Figure 1.**
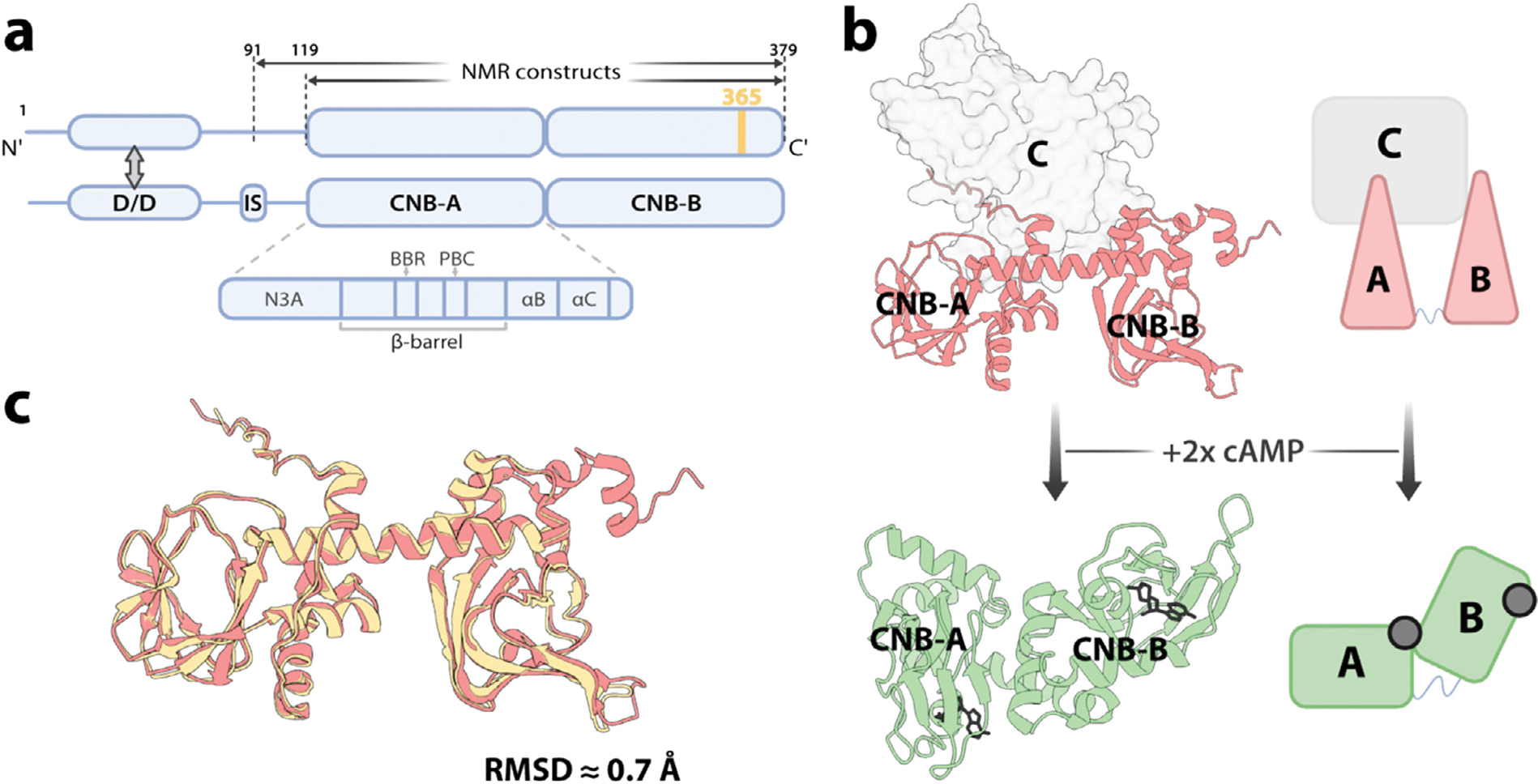
Architecture of PKA RIα. **(a)** Structural overview of the PKA RIα homodimer and constructs used. A gray double arrow indicates inter-monomer interactions at the dimerization-docking (D/D) domain. Each C-terminal tandem cyclic-nucleotide binding (CNB) domain binds one cAMP equivalent through a base binding region (BBR), and a phosphate binding cassette (PBC). The 119–379 construct spans the two CNB domains, while the 91–379 fragment includes the inhibitory site (IS) responsible for auto-inhibition of the kinase subunit. A yellow line indicates the site of the R366X ACRO truncation mutant. **(b)** 3D structure and cartoon representation of the C-subunit bound inactive PKA heterodimer (salmon) and cAMP-bound active RIα monomer (green). The C-subunit and cAMP are shown in light gray surface and black, respectively. **(c)** Overlay of C-subunit bound WT (2QCS [6]) and R366X (5JR7 [22]) inactive X-ray crystal structures in salmon and yellow respectively. C-subunits were removed from the inactive heterodimer structures for clarity, and their RMSD difference was calculated by aligning the two structures in PyMOL.

cAMP-dependent Protein kinase A (PKA) is a ubiquitous and vital molecular sensor of cyclic adenosine monophosphate (cAMP) and controls a plethora of signaling pathways that amplify extracellular stimuli from primary messengers (*e.g*. hormones), into tightly controlled intracellular responses [1,2]. PKA is comprised of separately encoded catalytic (C) and regulatory (R) subunits existing in an auto-inhibited complex (holoenzyme) state consisting of two C-subunits bound to an R-subunit homodimer [3–6]. Within the complex, each R is individually bound to and inhibits one of the two C-subunits (Figure 1). Each R-subunit comprises a multidomain protein composed of an N-terminal dimerization/docking (D/D) domain, a disordered N-terminal linker and two cAMP binding domains (CNB-A and CNB-B) (Figure 1a) [6]. The CNBs are conserved cAMP receptors comprised of a central β-sandwich motif, with an embedded phosphate binding cassette (PBC), an N-terminal helix-turn-helix (N3A) motif, and two C-terminal α-helices (αB and αC; Figure 1a) [4,6,7].

Binding of cAMP to the R-subunit CNB domains enables the unleashing of C and activation of the kinase (Figure 1b) [6,8]. Here, cAMP first binds to the more exposed CNB-B, enabling recruitment of the CNB-A capping residue (W260) which stabilizes the binding of cAMP in domain A via π-π stacking interactions [6,9–11]. The CNB-B domain is therefore known as the gatekeeper to activation. By requiring a sequential allosteric mechanism to occur before full subunit dissociation, CNB-B serves to regulate PKA and prevent inefficient kinase activation [6,11]. Hydrolysis of cAMP to 5′-AMP by phosphodiesterases (PDEs) in turn allows for termination of the cAMP signal cascade [12–14]. Due to the observed lower cAMP residence time in CNB-A, the deactivation mechanism has been hypothesized to occur though an intermediate with a cAMP-bound CNB-B and an unbound CNB-A [8,15]. According to this hypothesis, the sequential cAMP-PKA signal termination mechanism therefore mirrors that of activation but in reverse order. Full inhibition is achieved upon removal of cAMP from both CNBs [15,16]. The balance of cAMP synthesis by adenylyl cyclases (ACs) and hydrolysis by PDEs, together with subcellular localization, allows for tight spatio-temporal regulation of cAMP signaling.

Mutations in RIα that alter the activation and/or termination of the cAMP-signal lead to aberrant PKA function [11,15]. For example, inherited RIα mutations that make PKA resistant to cAMP activation cause PKA hypoactivation and are implicated in Acrodysostosis (ACRO) – a pathological condition characterized by congenital malformations and potentially severe developmental delays [17–23].

Here we focus on the most recurrent and severe ACRO mutant [20,21,24–31], *i.e*. the R366X nonsense mutation, which causes a 14-residue truncation of the C-terminal CNB-B of RIα. The crystal structure of a monomeric R366X RIα mutant has been solved in complex with the catalytic subunit, leading to the identification of key sites responsible for preventing proper kinase activation [22]. However, this structure only captures the inhibited and most stable state of the PKA RIα mutant, and shows only marginal differences from the wild type structure (RMSD ~ 0.7 Å; Figure 1c). Additionally, previous studies have only examined the effects of ACRO mutations on the activation cycle of PKA (*i.e*. sensitivity to cAMP binding) as opposed to PKA deactivation in ACRO variants. In this work, we focus on the less understood signal termination cycle and extend the current understanding of R366X also by examining the cAMP-bound conformation in solution.

We hypothesize that the R366X ACRO RIα mutation acts not only by disrupting binding to CNB-B but also by affecting the interdomain allosteric networks of RIα. We also hypothesize that the R366X ACRO RIα mutation alters cAMP signaling by eliciting a two-pronged effect, *i.e*. by partially impeding PKA activation and by accelerating the deactivation pathway as a result of shortened cAMP signal lifetimes and enhanced effective rates of cAMP hydrolysis by PDEs. To test our hypotheses, we relied on real-time NMR to monitor the effects of the PDE-catalyzed hydrolysis of cAMP at residue resolution as well as on circular dichroism (CD), fluorescence, and unfolding free energy measurements. Our results show that the R366X ACRO mutation allosterically accelerates cAMP dissociation at both tandem CNB domains, reverses the canonical deactivation cycle of PKA, and destabilizes the structural integrity of RIα. These findings highlight severe long-range effects induced by the R366X ACRO mutation and reveal a novel pathological mechanism of ACRO mutations.

## Results

### The R366X mutation reverses the order of cAMP release and accelerates PKA deactivation

**Figure 2.**
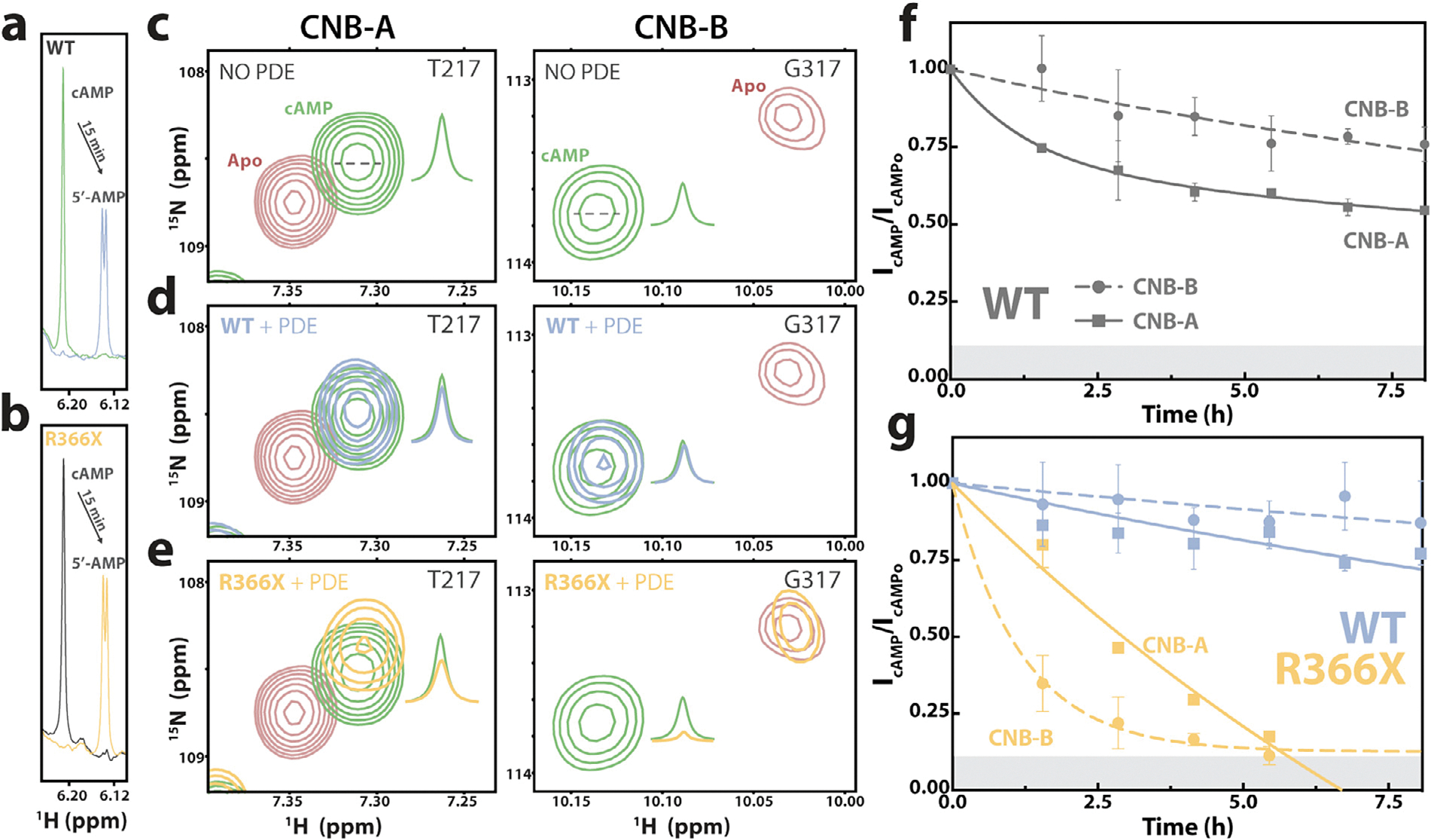
Monitoring the PDE-catalyzed hydrolysis of cAMP by real-time NMR reveals that the ACRO1 R366X mutant accelerates and reverses the PKA deactivation pathway. (**a**) 1D ^1^H H1′ signal of cAMP and 5′-AMP used to monitor the hydrolysis of 100 μM free excess cAMP (green) after 15 min (blue) in the presence of 20 nM PDE and 40 μM WT (119–379). (**b**) As (**a**) using 40 μM R366X (119–365) instead. (**c–e**) Selected regions of 2D ^1^H–^15^N HSQC spectra of ^15^N-labeled PKA RIα acquired during the PDE-catalyzed cAMP hydrolyses using sample starting conditions as in (a). Representative cross-peaks for CNB-A and -B are shown for cAMP-bound WT (green, c) and apo WT (red, c) as reference spectra. Representative CNB-A and -B cross-peaks for spectra of cAMP-bound WT (light blue, d) and R366X (yellow, e) in the presence of 20 nM PDE after 1.5 h. Gaussian fitted 1D cross-sections highlight the decay of the cAMP-bound peak. (**f**) Loss of CNB-A and B-bound cAMP in the presence of 60 nM PDE and 40 μM WT RIα, monitored via residues T217 & T207 (CNB-A, solid line), and G314, G317, and A339 (CNB-B, dotted line). (**g**) as (**f**) but for the cAMP hydrolysis in the presence of 20 nM PDE and 40 μM WT or R366X RIα. Error bars are derived by propagating errors assessed as standard deviation between residues. The gray box at the bottom represents the average noise level in the HSQC spectra.

To gauge how R366X affects the PKA deactivation mechanism after cAMP-dependent activation, we monitored the kinetics of cAMP removal from each CNB domain through 2D ^1^H–^15^N heteronuclear single quantum coherence (HSQC) spectra of ^15^N-labeled RIα (119–379) acquired in real-time after addition of sub-stoichiometric amounts of PDE. Supplementing the reaction mixtures with 100 μM excess free cAMP prior to PDE addition ensured that the CNB domains were still cAMP-bound after the dead-time elapsed between PDE addition and the start of the acquisition for the first HSQC spectrum. In all reactions, the 100 μM excess unbound cAMP was completely hydrolyzed to 5′-AMP within 15 min (Figure 2a,b), at which point any subsequent cAMP hydrolysis arose solely from the release of cAMP from RIα given that RIα protects cAMP from PDEs.

To examine the release of cAMP from the CNB domains, we selected specific residues in each domain that experience significant chemical shift changes between their cAMP-bound and cAMP-unbound (Apo) states to provide sufficient peak resolution (Figure 2c). Since for RIα the on–off exchange of cAMP is slow in the chemical-shift timescale [32], the decrease in the intensity of cAMP-bound RIα peaks over time after PDE addition was quantified for the WT and R366X using these peaks (Figure 2d,e). Our results for WT RIα (119–379) in the presence of 60 nM PDE show that cAMP is removed more quickly from CNB-A than from CNB-B (Figure 2f), consistent with the previously reported shorter residence time of cAMP in WT CNB-A *vs*. WT CNB-B [8]. Using the same concentration of PDE with R366X RIα, however, caused loss of cAMP from both domains in a timescale too short for acquiring HSQC spectra with sufficient S/N ratios and resolution (data not shown). Hence, the PDE concentration was reduced three-fold to monitor relative losses of RIα-bound-cAMP at domain resolution. The reduced PDE concentration allowed us to observe in real-time the cAMP-signal termination pathway also for the R366X mutant, revealing an alternative mode of deactivation clearly distinct from WT RIα. Unlike WT RIα, in the R366X mutant cAMP removal occurs preferentially at CNB-B followed by CNB-A – a reversal of the order of cAMP release compared to WT RIα (Figure 2f,g). Additionally, our results show an overall acceleration of cAMP removal from both domains in the R366X mutant relative to WT. These findings suggest that the 366X truncation not only increases the *k*_off_ rate of CNB-B-bound cAMP (*i.e*. decreases its residence time) but also causes long-range perturbations on CNB-A that facilitate the release of CNB-A-bound cAMP.

### Mutated CNB-B domain experiences a shortened cAMP residence time

**Figure 3.**
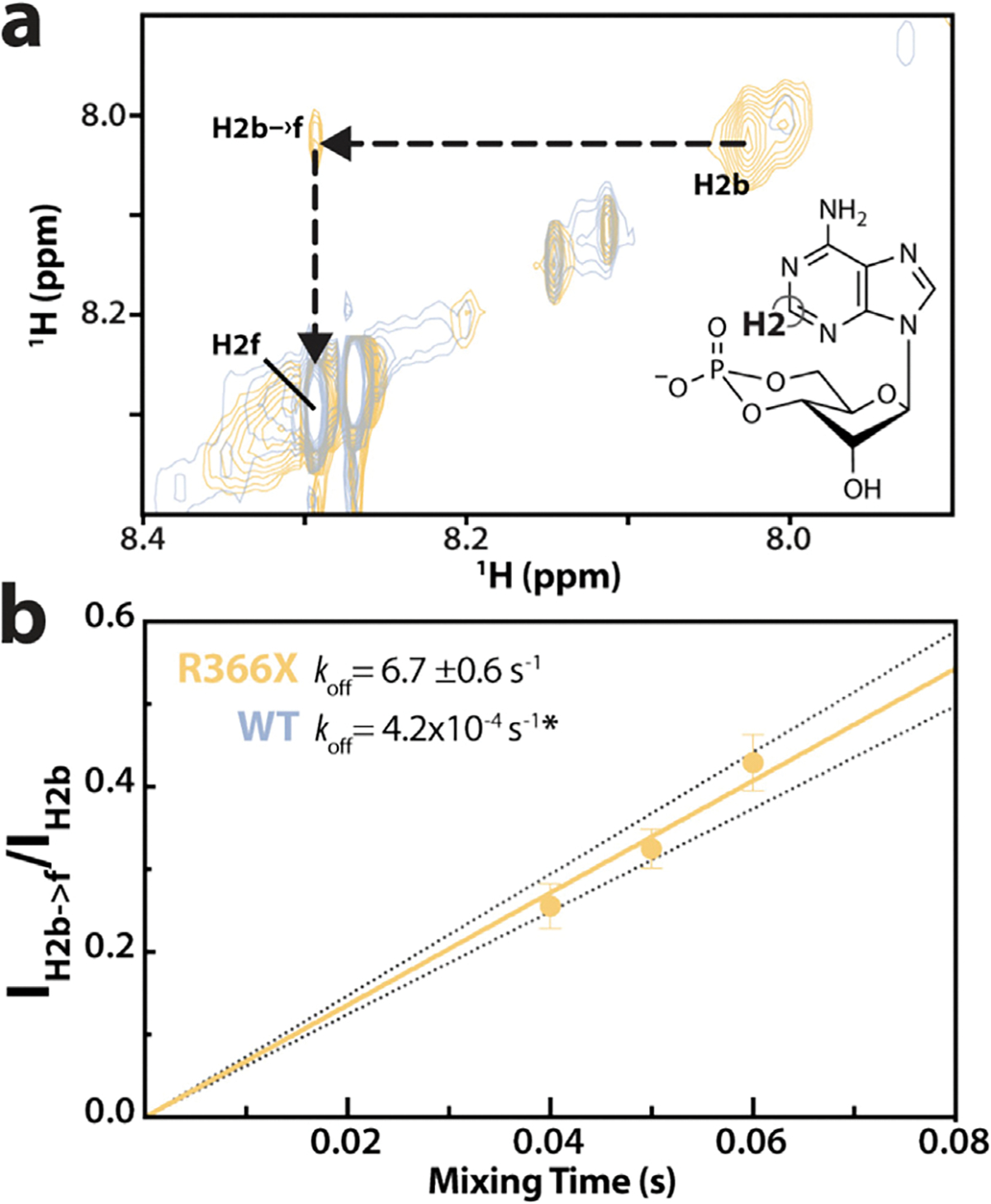
ACRO1 366X reduces the residence time of cAMP bound to CNB-B. **(a)** Overlaid NOESY spectra of WT (gray) and R366X (yellow) RIα in the presence of 500 μM cAMP. The structure of cAMP highlighting the H2 position is shown as inset. The cross-peak (H2b → f) arises from chemical exchange of cAMP from the bound state (H2b) to the free state (H2f). **(b)** Plot of the H2b → f cross-peak to the H2b diagonal peak intensity ratios against the NOESY mixing time, yielding the dissociation rate constant (*k*_off_) of cAMP from CNB-B. The error was calculated by propagation of signal-to-noise ratios. The “*” indicates that the *k*_off_ of cAMP bound to WT CNB-B is too low to be measured through NOESY spectra but was previously reported in the literature [8,51].

To test our hypothesis that the R366X mutation decreases the residence time of CNB-B-bound cAMP, nuclear Overhauser effect spectroscopy (NOESY) experiments were employed to directly measure the rate of exchange of cAMP from the R366X RIα-bound to the free state. While the NOESY spectra for WT RIα (Figure 3a, gray) showed no detectable cross-peak (*i.e*. no bound-to-free transition), as expected based on the low-nM affinity of WT for cAMP and its residence times of 1–45 min [33,34], the NOESY spectra of the R366X mutant showed a clear exchange cross-peak for the proton at position 2 (H2) of the nucleobase moiety of cAMP (Figure 3a, yellow and insert). This is consistent with a faster off rate at CNB-B, which is likely caused by the absence of the cAMP capping residue of CNB-B (*i.e*. Y371) — responsible for stabilizing CNB-B-bound cAMP via π-π stacking interactions in WT RIα. This NOESY cross-peak arises from the exchange of cAMP from the bound to the free state (H2b → f), yielding a residence time (1/*k*_off_) of cAMP in the mutate d CNB-B in the sub-second timescale (Figure 3b). This is a drastic reduction of ~four orders of magnitude compared to the timescale of cAMP release from WT CNB-B, which is in the order of tens of minutes [8]. These findings confirm that the R366X truncation mutant drastically accelerates the dissociation of cAMP from CNB-B.

### Long-range allosteric effects elicited by the R366X mutation in CNB-B extend to CNB-A

**Figure 4.**
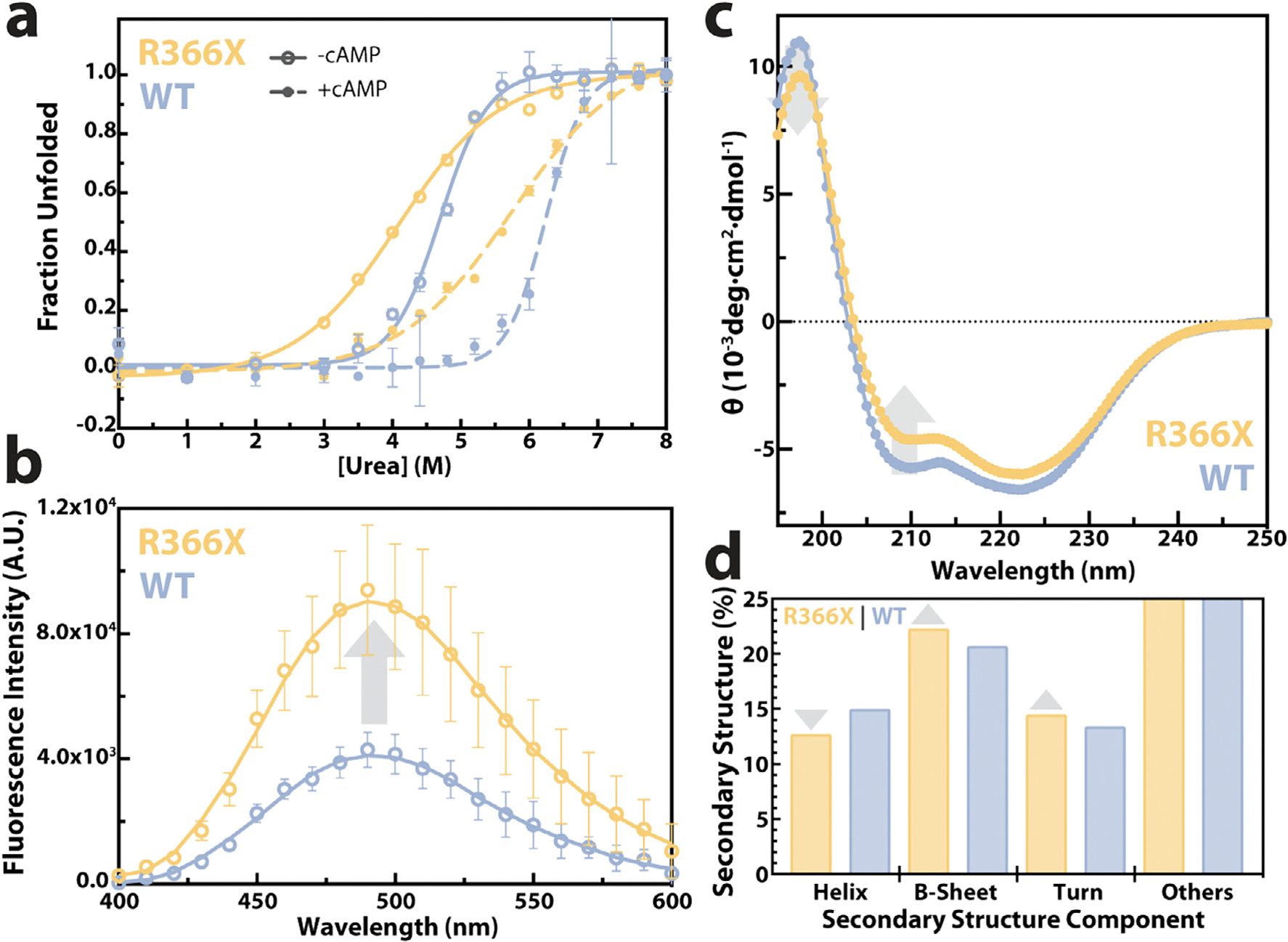
R366X in CNB-B allosterically destabilizes CNB-A. **(a)** Urea unfolding of 5 μM WT (119–379, gray) and R366X (119–365, yellow) with and without 100-fold excess cAMP. **(b)** ANS fluorescence spectra and **(c)** Far-UV CD spectra of WT and mutant RIα. (d) Predicted secondary structure composition of WT and R366X RIα computed using the Beta Structure Selection (BeStSel) algorithm [46,47]. Values for the off-scale ‘Others’ category are 51% for both R366X and WT.

To explain how the R366X CNB-B mutation additionally influences CNB-A, we first took a low-resolution approach via urea denaturation assays. We took advantage of intrinsic tryptophan fluorescence from three Trp residues whithin RIα, which are spatially confined primarily to CNB-A and report on allosteric effects caused by the CNB-B truncation [35,36]. The comparative analysis of the unfolding profiles monitored by Trp fluorescence for 366X *vs*. WT RIα (Figure 4a) indicates that the R366X mutation induces long-range destabilizing effects on the CNB-A domain. The R366X mutant not only exhibits a lower unfolding midpoint urea concentration (*C*_*m*_), but also results in a shallower slope (*m*; Table S1 and Figure 4a), pointing to divergence from the sharper two-state transition observed for WT RIα, and to an increase in the solvent-accessible surface area of the mutant [37]. To corroborate this result, we complemented the urea denaturation results (Figure 4a) with 1-Anilino-8-napthalenesulfonate (ANS) fluorescence experiments (Figure 4b), which report on exposure of hydrophobic regions [38,39]. The ANS data show increased fluorescence in R366X compared to WT (Figure 4b), confirming that the mutation causes increased solvent exposure relative to WT RIα. These findings are consistent with CD spectral changes in regions that report on α-helicity (Figure 4c), indicating a decrease in α-helical populations and an increase in the proportion of more extended and turn elements (Figure 4d).

### Residue-specific chemical shift changes confirm that R366X perturbs the cAMP binding sites in both CNB-A and B

**Figure 5.**
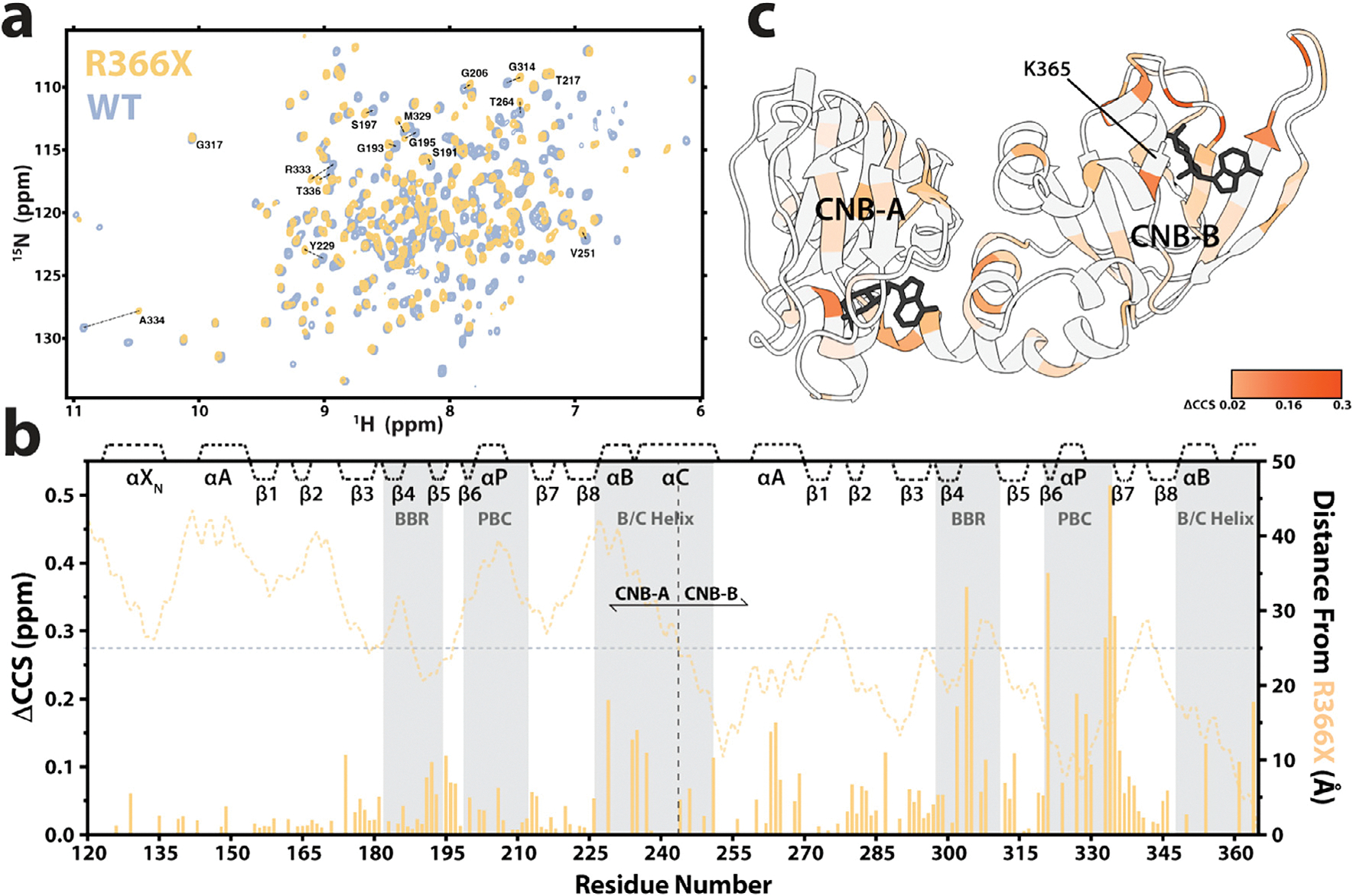
R366X in CNB-B perturbs chemical shifts in CNB-A. **(a)** ^1^H–^15^N TROSY spectra of cAMP-bound 91–379 (gray, WT) and 91–365 (yellow, R366X). **(b)** Residue-specific compounded chemical shift differences (ΔCCS) between WT and R366X. The dotted yellow line shows the distance of each residue from the C-terminal residue 365. **(c)** ΔCCS above the average of residues further than 25 Å (horizontal dotted line) from the mutation site, visualized on the structure of cAMP-bound RIα via ChimeraX (1RGS [52]).

We employed 2D ^1^H–^15^N transverse relaxation optimized spectroscopy (TROSY) NMR experiments (Figure 5a) to examine the effects of R366X at residue resolution. For these studies that don’t require time-resolution and fast acquisition, we utilized the longer cAMP-bound 91–379 (or 91–365) RIα construct, which includes part of the N-terminal linker and the C-subunit inhibitory site. The unstructured nature of this linker, however, caused some peak overlap and thus we chose to focus on well-resolved peaks of the region spanning the two CNB domains (residues 119–365) for our analyses (Figure 5b). The compounded chemical shift differences (ΔCCS) between cAMP-bound-WT and R366X were used to identify perturbations caused by the mutation. As expected, we detected major chemical shift caused by the R366X mutation in CNB-B, the site of the truncation (Figure 5b,c). These ΔCCS appear to concentrate around key regions responsible for cAMP binding, namely the PBC (Figure 5b,c), and the base-binding region (BBR, Figure 5b,c). Additionally, significant chemical shift changes are observed to extend past CNB-B, affecting the B/C helices connecting the two domains and into CNB-A (Figure 5c). Within the CNB-A domain, once again the ΔCCS arise around the PBC and BBR regions, suggesting that R366X influences cAMP-binding interactions not only within CNB-B but also in the neighboring CNB-A domain.

We also conducted chemical shift projection analyses (CHESPA), a tool that compares two reference states (*e.g*. cAMP-bound and apo) to a third state (*e.g*. a mutant; Figure S1a,b insert) [40]. The CHESPA for residues with assignments available for the three different states (*i.e*. RIα 91–379 apo and cAMP-bound, and 91–365 cAMP-bound; Figure S1a–d) revealed that both CNB domains are subject to a predominant shift towards the inactive-like apo state (Figure S1a, b), as supported by the higher proportion of negative values (Figure S1d). The concurrent presence of some residues exhibiting shifts toward the active state indicates that the effect of the R366X mutation is not fully recapitulated by a simple two-state shift towards inactivation. Nevertheless, the overall CHESPA results suggest that the R366X mutant causes an at least partial shift to inactive-like states in each domain, even in their cAMP-bound form.

## Discussion

**Figure 6.**
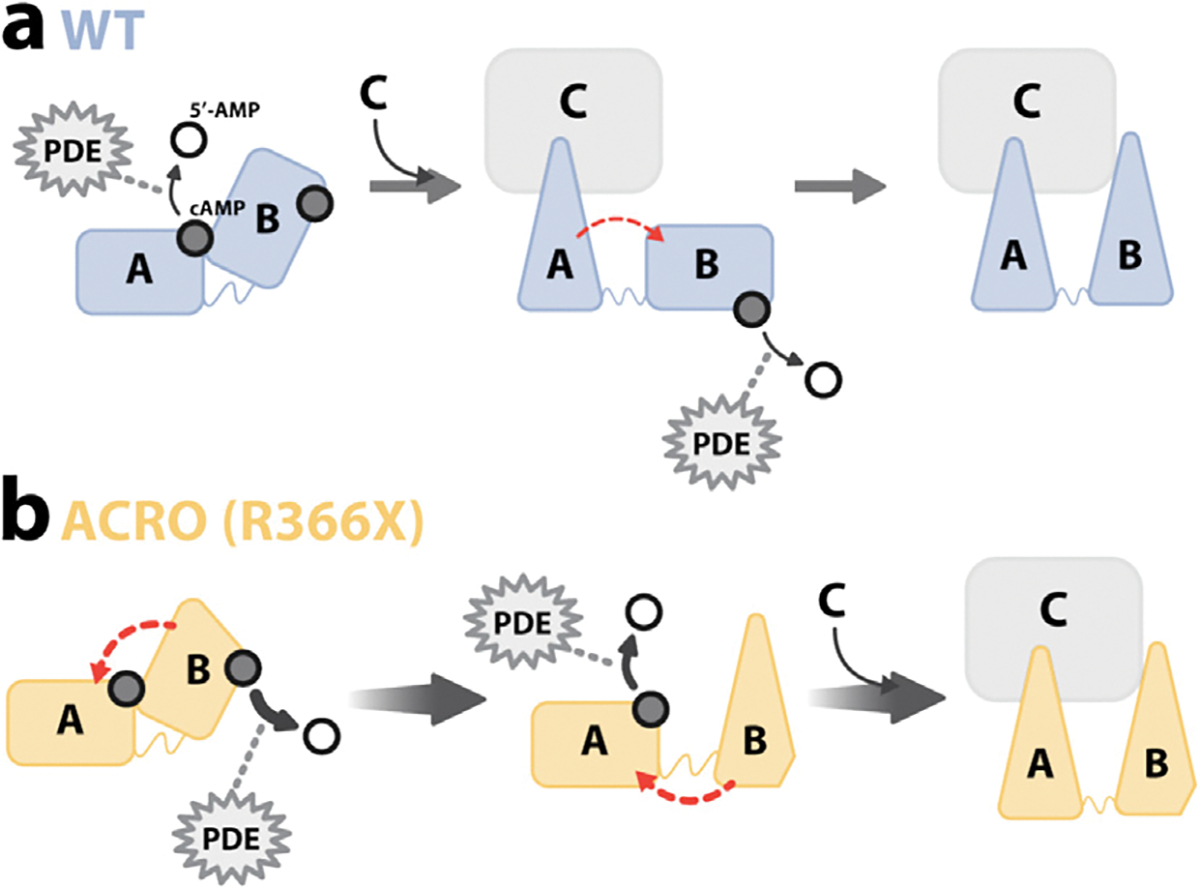
Novel mechanism of deactivation proposed for the PKA RIα R366X ACRO1 mutant. Deactivation mechanisms of (**a**) WT and (**b**) ACRO1. Active and inactive CNB domains are represented by rectangles and triangles, respectively. cAMP and 5′-AMP are shown as black filled and empty circles, respectively. A faster rate of release from CNB domains is indicated by increasing thickness of curved black arrows, while the thicker gradient arrows between states emphasize the accelerated rate observed for the deactivation of ACRO1 RIα 366X. Dashed curved red arrows represent inter-CNB domain allosteric effects.

So far, ACRO1 mutations have been examined primarily from the lens of kinase activation, whereby these mutations often decrease the binding affinity for cAMP causing resistance to cAMP activation. In our study, we take a complementary approach by focusing on the PKA deactivation mechanism, and assessing the effects of the R366X mutation on the signal termination cycle. Our results on WT RIα provide an unprecedented residue-resolution picture of PKA deactivation, whereby cAMP dissociates preferentially from RIα CNB-A than CNB-B, leading to the re-formation of a fully inhibited holo complex between the C-subunit and RIα (Figure 6a) [6,8,15]. This partially sequential model of WT RIα deactivation is consistent with the notion that RIα-bound cAMP is protected from PDEs and that CNB-A-bound cAMP exhibits a õne-order-of-magnitude shorter residence time than CNB-B-bound cAMP [15,41,42]. We show that ACRO1 mutations alter this canonical mechanism of deactivation in two key respects. First, our results reveal a cAMP-signal-termination mechanism for 366X RIα that is reversed compared to WT RIα with cAMP being preferentially released from CNB-B *vs*. CNBA (CNB-B → CNB-A). This results in the formation of a deactivation intermediate with cAMP bound to CNB-A and apo CNB-B of RIα (Figure 6b). Second, the 366X mutation accelerates cAMP removal from both CNB domains compared to WT (Figure 6b). This means that, while removal of cAMP from CNB-B alone is insufficient for PKA inactivation, it leads to the hastened removal of cAMP from CNB-A, which results in PKA inhibition (Figure 6b).

The effect of the 366X mutation on the kinetics of cAMP signal termination arises at least in part from the fact that cAMP bound to CNB-B of R366X exhibits a four-orders-of-magnitude shorter residence time relative to WT, explaining its faster dissociation and hydrolysis in the presence of PDE. While this accelerated loss of cAMP from the mutated CNB-B was foreseeable, the effects of this mutation on CNB-A were not. Our urea unfolding and chemical shift data indicate that R366X causes global allosteric effects on CNB-A and show that these effects persist in the cAMP-bound form. While CD data show that R366X remains overall structured, urea-induced unfolding and ANS fluorescence suggest global instability and a more solvent-exposed conformation. These results point towards a dynamic mutant structure with perturbed cAMP interactions even in the distal CNB-A. Our NMR results corroborate this interpretation, showing chemical shift changes in the CNB-A domain, including key cAMP-binding regions, and the B/C helices of truncated RIα. Hence, the partially sequential release of cAMP from the tandem domains arises from a combination of inter-domain allostery and differences in *K*_*d*_ and *k*_off_ values for cAMP binding to the two domains.

Based on our data, we propose a model where the ACRO1 CNB-B mutation 366X contributes cAMP-destabilizing effects to CNB-A in two ways. First, when RIα is fully bound to cAMP, the R366X mutation in CNB-B allosterically perturbs CNB-A: cAMP contacts causing at least partial cAMP dissociation. Second, once cAMP is released from CNB-B, these effects are amplified and enhanced by a shift of CNB-B to inhibitory states, which disrupts the inter-domain interface [10]. This non-canonical mechanism significantly accelerates kinase deactivation, suggesting that even when ACRO1 mutants bind cAMP, fast cAMP dissociation and hydrolysis lead to premature PKA deactivation. This model, therefore, offers a novel explanation for the typical ACRO1 clinical phenotype of hormonal resistance.

## Materials and Methods

### Protein expression and purification

All proteins were expressed in the *E. coli* BL21 (DE3) strain and resuspended cells were lysed in lysis buffer using a continuous flow cell disruptor (Constant Systems) at 20 kpsi. Protein concertation was quantified via the Bradford colorimetric assay and purity was assessed via sodium dodecyl sulfate gel electrophoresis (SDS-PAGE). Mutations in PKA RIα, including the truncation mutant constructs (91–365 and 119–365), were prepared via site-directed mutagenesis using in-house designed primers and the KOD One PCR Master Mix (Millipore Sigma), followed by digestion using the DpnI restriction enzyme (ThermoFisher) and *in vivo* assembly by transformation into One Shot TOP10 Chemically Competent *E. coli* (ThermoFisher).

The PKA RIα (119–379 and 119–365) two-domain constructs were prepared as previously outlined [42], except for the following steps. The protein was overexpressed in either LB medium for non-NMR experiments, or in M9 minimal medium supplemented with ^15^N-labeled NH_4_Cl for NMR experiments. Expression was induced using 0.5 mM isopropyl ß-D-1-thiogalactopyranoside (IPTG) at 20 °C for ~ 20 h. Cell pellets were resuspended in a MES-based lysis buffer [20 mM 2-(N-morpholino)ethanesulfonic acid (MES) (pH 6.5), 100 mM NaCl] prior to lysis. After protein precipitation by ammonium sulfate, the isolated precipitate was resuspended in a MES-based buffer [20 mM MES (pH 6.5), 100 mM NaCl, 2 mM ethylene glycol tetra-acetic acid (EGTA), 2 mM ethylenediaminetetraacetic acid (EDTA), 5 mM dithiothreitol (DTT)]. The PKA RIα (91–379 and 91–365) constructs were prepared by following prior protocols established for the 91–244 PKA RIα construct [43]. The PDE8A1 catalytic domain (472–829) was prepared as previously described [16]. After separation by size-exclusion chromatography (SEC), only the dimer form was selected for use in the PDE-catalyzed hydrolysis reactions [44].

### NMR spectroscopy

NMR spectra were collected at 306 K on a Bruker Avance NEO 700 MHz spectrometer equipped with a TCI cryo-probe, unless otherwise stated. Processing was implemented using Bruker’s TopSpin 4.4.0, and spectral analysis was conducted via POKY. Spectral assignments of R366X mutant constructs were obtained via spectral comparisons, excluding any ambiguous residue assignment.

### Monitoring of domain-specific PDE-catalyzed cAMP hydrolysis by real-time NMR

NMR spectra were collected at 306 K on a Bruker Avance III HD 850 MHz spectrometer equipped with a TCI cryo-probe. 1D ^1^H spectra were acquired with a total of 64 scans, using 32 K complex points and a spectral width of 15.9 ppm centered at 4.7 ppm. 2D ^1^H–^15^N HSQC NMR spectra were acquired with 64 scans, using 2 K and 128 complex points and spectral widths of 15.98 ppm and 35 ppm centered at 4.7 ppm and 117 ppm in the ^1^H and ^15^N dimensions, respectively. The spectra were processed with a squared sine bell window function shifted by 60° for increased resolution. Reference spectra were acquired for 40 μM PKA RIα (119–379 or 119–365) with 100 μM excess cAMP, in MES-based buffer [50 mM MES (pH 6.5), 100 mM NaCl, 2 mM EDTA, 2 mM EGTA, 10 mM DTT, 10 mM MgCl_2_, 5% D_2_O]. cAMP hydrolysis in the presence of 20 or 60 nM PDE8A1 were then monitored overnight, acquiring 1D ^1^H and 2D ^1^H–^15^N spectra in sequence for a total of ~8 h. Loss of RIα-bound cAMP over time from each cAMP-binding domain was monitored by averaging the intensity ratio (*I*_cAMP_/*I*_cAMPo_) of peaks in the cAMP-bound conformation, using well-resolved assigned cross-peaks of CNB-A (T207 and T217) and CNB-B (G314, G317, and A339). Error bars represent the standard deviation between the averaged peak intensity ratios. To account for dilution of the reference sample, the 2D ^1^H–^15^N peak intensities were normalized using the ratio of the average 1D ^1^H peak intensities across the methyl and amide regions before and after PDE addition.

### Measurement of dissociation rate constant (k_off_) by NOESY experiments

NOESY spectra were collected on samples of 50 μM PKA RIα (119–379 or 119–365) with 500 μM cAMP in 20 mM NaH_2_PO_4_ (pH 6.5), 100 mM NaCl in ≥99% D_2_O. The spectra were acquired with 64 scans, a spectral width of 12 ppm in both ^1^H dimensions, a recycle delay of 1.2 s, and 4 K and 512 complex points in t_2_ and t_1_, respectively. Spectra were processed using a 90° shifted squared sine bell window function in both dimensions, with the first point scaled by 0.5 in the ^1^H dimension. NOESY spectra were acquired at three different NOE mixing times (40, 50, and 60 ms) for R366X, and one WT control spectrum was acquired with a mixing time of 40 ms. The *k*_*off*_ was measured through the slope of the intensity ratio of the bound-to-free (*I*_H2b→f_) NOE cross-peak to the protein-bound ligand (*I*_H2b_) diagonal peak, plotted against mixing times. The error bars were calculated by error propagation of the measured signal-to-noise ratios.

### Urea unfolding assays monitored via tryptophan fluorescence

PKA RIα (119–379 or 119–365) at a concentration of 5 μM with or without 100-fold excess of cAMP was incubated with different concentrations of urea (0–8 M) for 3 h at room temperature in MES-based buffer [50 mM MES (pH 6.5), 100 mM NaCl, 2 mM EDTA, 2 mM EGTA, 5 mM DTT]. Tryptophan fluorescence was measured on a Biotek Cytation 5 microplate reader with an excitation wavelength of 293 nm and emission wavelengths of 340 nm and 353 nm. Unfolding was monitored by using the ratio of fluorescence intensities at 340/353 nm. The fraction of protein unfolded ($F_{U}$) was calculated as in Eq. (1), where R is the observed intensity ratio at a given concentration of urea, while $R_{F}$ and $R_{U}$ are the averaged values of the plateaus of the fully folded and unfolded states, respectively [35,45]:

(1) $$F_{U}=1-\left[\frac{\left(R\;-\;R_{U}\right)}{\left(R_{F}\;-\;R_{U}\right)}\right]$$

### ANS fluorescence

ANS fluorescence data were collected using a final PKA RIα (119–379 or 119–365) concentration of 8 μM in MES-based buffer [50 mM MES (pH 6.5), 100 mM NaCl, 2 mM EDTA, 2 mM EGTA, 5 mM DTT] and 200 μM ANS. Fluorescence was measured at room temperature using a BioTek Cytation5 microplate reader with excitation at 350 nm and emission range of 400–700 nm. Data was acquired in triplicate for each sample, including a control of ANS alone. The negative control was subtracted at each wavelength. Errors were calculated by propagation of the standard deviation between triplicates.

### CD measurements and analysis

CD spectra were acquired using a JASCO J-1000 CD spectrophotometer on samples of 0.2 mg/mL PKA RIα (119–379 or 119–365) in 20 mM NaH_2_PO_4_ (pH 6.5), 100 mM NaCl. Spectra were recorded in a 0.1 cm cuvette between 190 and 260 nm at 50 nm/min, with a 2 s integration time, at room temperature, and for three accumulations. Data processing was conducted using the JASCO Spectra Manager^™^ software. First, a blank buffer spectrum was subtracted, followed by smoothing via the Savitzky-Golay method with a convolution width of 25 points. The data was then converted to mean residue molar ellipticity (θ in deg·cm^2^ dmol^−1^) via Eq. (2). Where ellipticity is the CD spectrophotometer output in mdeg units, l is the path length of the cuvette (mm), c is the concentration of RIα (μM), and n is the number of residues. Finally, the CD profiles were baseline corrected by subtracting the average θ of the plateau from 245 to 260 nm.


(2) $$\theta =\frac{\left(\text{ellipticity}\;\cdot \;10^{6}\right)}{I\;\cdot \;c\;\cdot \;n}$$

Secondary structure composition was obtained from the CD spectra using the Beta Structure Selection (BeStSel) algorithm [46,47]. The wavelength range of 195–250 nm was used due to potential buffer contributions at lower far-UV wavelengths [48]. Parallel and antiparallel β-sheet propensities were combined into a single value labeled as “β-sheets”.

### NMR chemical shift perturbation analyses

2D ^1^H–^15^N TROSY spectra of uniformly ^15^N-labeled PKA RIα (91–379 or 91–365) with partial deuteration at a concentration of 10 μM with 2.5 mM excess cAMP in MOPS-based buffer [50 mM 3-(N-morpholino)propanesulfonic acid (MOPS) (pH 7.0), 100 mM NaCl, 5 mM DTT] were acquired with 320 scans, using 2 K and 128 complex points, and spectral widths of 17.85 ppm and 36 ppm centered at 4.7 ppm and 117.9 ppm in the ^1^H and ^15^N dimensions, respectively. The spectra were processed using a 45° sine square window function. CHESPA was implemented as previously outlined [49], using previously measured apo chemical shifts [50]. The ΔCCS were calculated using equation (3), where $\delta_{H}$ and $\delta_{N}$ are the chemical shifts in the ^1^H and ^15^N dimensions, respectively.


(3) $$\Delta CCS=\sqrt{{\left(\delta_{H,1}-\delta_{H,2}\right)}^{2}+{\left(0.2\cdot \left(\delta_{N,1}-\delta_{N,2}\right)\right)}^{2}}$$

## Supplementary Material

supplementary material

Appendix A. Supplementary material

Supplementary material to this article can be found online at https://doi.org/10.1016/j.jmb.2025.169381.

## Data Availability

Data will be made available on request.

## Abbreviations:

PKA: cAMP-dependent protein kinase or protein kinase A
cAMP: cyclic adenosine monophosphate
C: catalytic subunit of PKA
R: regulatory subunit isoform Iα of PKA
D/D: dimerization/docking domain of RIα
CNB: cyclic-nucleotide binding domain of RIα
PBC: phosphate binding cassette within the CNB
N3A: N-terminal helix-turn-helix motif within the CNB
PDE: phosphodiesterase (in this work the PDE8A1 catalytic domain)
ACRO: acrodysostosis
RMSD: root mean square deviation
CD: circular dichroism
HSQC: heteronuclear single quantum coherence
NOESY: nuclear Overhauser effect spectroscopy
ANS: 1-anilino-8-napthalenesulfonate
TROSY: transverse relaxation optimized spectroscopy
ΔCCS: compounded chemical shift differences
BBR: base binding region within the CNB
CHESPA: chemical shift projection analysis
