# Supplementary material for: Recurrent Acrodysostosis-Related PKA RIα Mutant Reveals a Novel Mechanism of Aberrant PKA Deactivation
Source: J Mol Biol. Author manuscript; Available in PMC 2026 Jan 28. (PMC12851291; doi:10.1016/j.jmb.2025.169381)
Supplement: supplementary material [file NIHMS2121122-supplement-supplementary_material.docx]

**Supplementary Material**

**for**

Recurrent Acrodysostosis-Related PKA RIα Mutant Reveals a Novel Mechanism of Aberrant PKA Deactivation

Leonardo Della Libera^a^, Karla Martinez Pomier^a^, Madoka Akimoto^a^, Ganesh S. Anand^b^, Susan S. Taylor^c^, Giuseppe Melacini*^a,d^

^a^Department of Chemistry and Chemical Biology, McMaster University, Hamilton, ON, Canada.

^b^Department of Chemistry, Department of Biochemistry and Molecular Biology, and The Huck Institutes of the Life Sciences, The Pennsylvania State University, University Park, PA 16802, USA.

^c^Department of Pharmacology and Department of Chemistry and Biochemistry, University of California, San Diego, La Jolla, CA 92037-0654, USA.

^d^Department of Biochemistry and Biomedical Sciences, McMaster University, Hamilton, ON, Canada.

*To whom correspondence should be addressed: [melacin@mcmaster.ca](mailto:melacin@mcmaster.ca)

**Table S1: Thermodynamic analysis of urea unfolding for WT and R366X PKA RIα.***

|  | non-excess cAMP | | | excess cAMP | | |
| --- | --- | --- | --- | --- | --- | --- |
| Protein | *C*_m_ (M) | *ΔG°*_H₂O_ (kcal mol^-1^) | *m* (kcal mol^-1^M^-1^) | *C*_m_ (M) | *ΔG°*_H₂O_ (kcal mol^-1^) | *m* (kcal mol^-1^M^-1^) |
| WT  (119-379) | 4.7 | 7.4 | -1.6 | 6.2 | 10.9 | -1.8 |
| R366X  (119-365) | 4.1 | 3.6 | -0.9 | 5.7 | 4.4 | -0.7 |
| *Urea unfolding data was acquired using 5 μM PKA RIα (119-379 or 119-365) and 100-fold excess cAMP. | | | | | | |

**
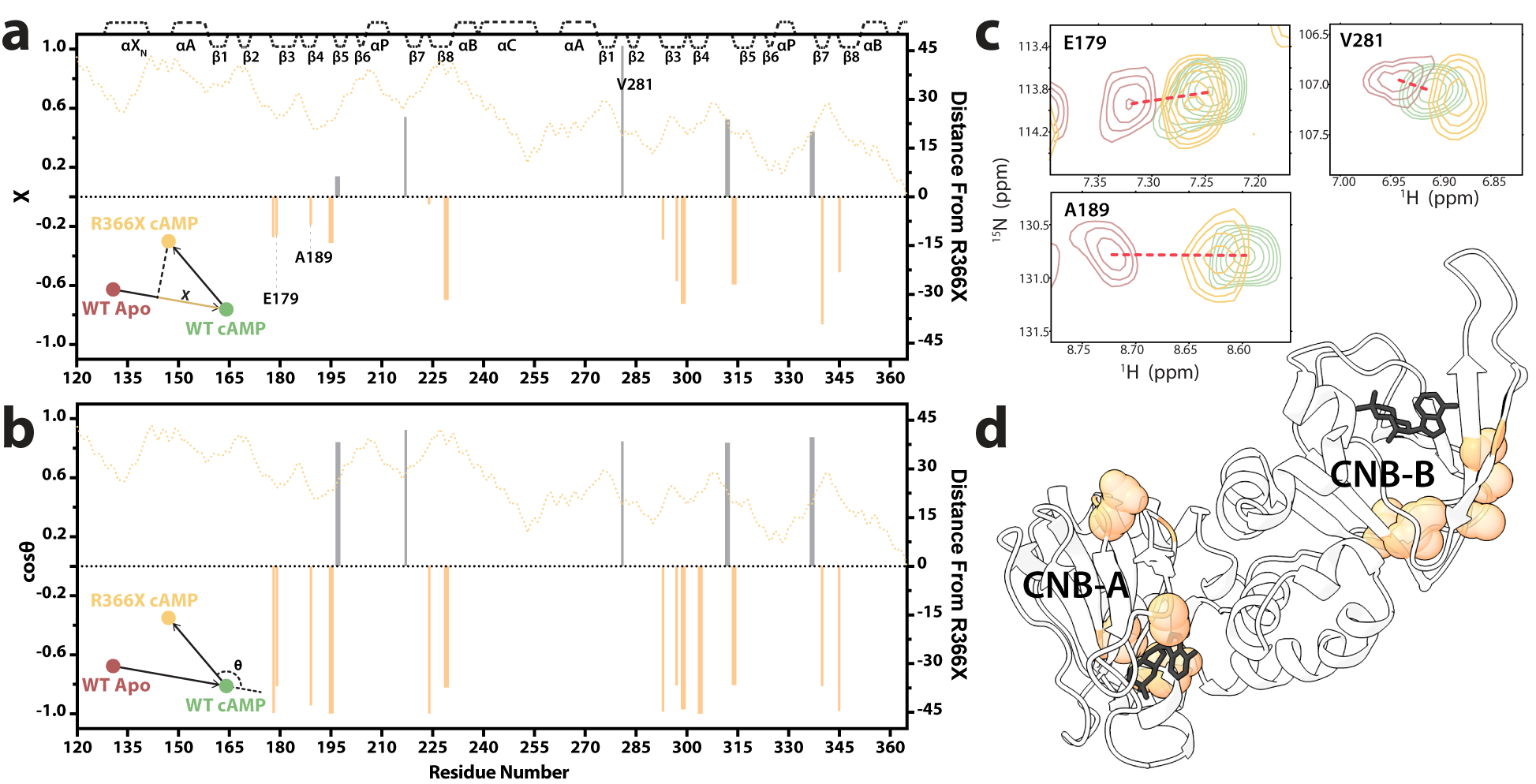
**

**Figure S1: *CHEmical Shift Projection Analysis (CHESPA) of R366X indicates that residues shifting towards inactive conformations prevail in both CNB-A and -B****.* (**a**) Fractional shift of R366X residues towards the WT active cAMP-bound state (gray bars), or inactive Apo state (orange bars). (**b**) cosθ *vs.* residue plot where θ is the projection angle. Both plots were constructed using a ppm cut off of 0.02 ppm (thin bars) or 0.05 ppm (thick bars) for the reference vector and excluding residues with a |cosθ| below 0.8 as they significantly deviate from linearity. (**c**) Representative ^1^H-^15^N TROSY cross-peaks of residues exhibiting a linear relationship between the three states. (**d**) 3D map of residues demonstrating a trend towards inactivation (orange) plotted on the structure of cAMP-bound PKA RIα (1RGS [52]). R366X and WT spectra are as in Fig. 5.


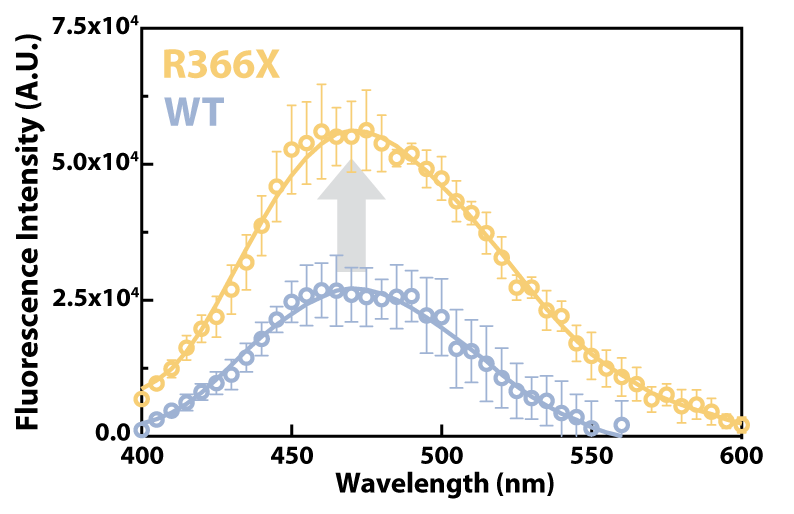


**Figure S2: ANS fluorescence spectra of WT and mutant RIα.** ANS data was acquired as in (Fig. 4b) except with 20 μM PKA RIα and 40 μM ANS.
